# 1801. About Damn Time: HAV, HBV, and Tetanus Screening and Vaccination during Hospitalization in Persons who Use Drugs

**DOI:** 10.1093/ofid/ofad500.1630

**Published:** 2023-11-27

**Authors:** Amber C Streifel, Jose E Rivera Sarti, Monica K Sikka, Michael Conte, Bradie Winders, Cara D Varley

**Affiliations:** Oregon Health and Science University, Portland, Oregon; Oregon Health Sciences University, Portland, Oregon; Oregon Health and Science University, Portland, Oregon; Oregon Health and Science University, Portland, Oregon; New York State Department of Health, Albany, New York; Oregon Health and Science University, Portland, Oregon

## Abstract

**Background:**

Rates of serious injection related infections (SIRI) in persons who use drugs (PWUD) have increased over the past two decades. Admissions for SIRI are an opportunity for screening and vaccination of preventable infections like hepatitis A virus (HAV), hepatitis B virus (HBV) and tetanus given significant barriers to preventive care in the outpatient setting for this population. The purpose of this study was to evaluate rates of screening and subsequent vaccination for hepatitis A, hepatitis B, and tetanus for PWUD admitted to the hospital for treatment of serous bacterial infection.

**Methods:**

We conducted a retrospective review of adult patients admitted for a bacterial infection requiring > 2 weeks of antibiotics with both infectious disease (ID) and addiction medicine consults. Data on patient demographics, screening recommendations, and testing and vaccination administration were collected via chart review.

**Results:**

280 patients were eligible for inclusion. Of the 198 (70.7%) patients determined at risk for HAV, ID recommended vaccination for 21 (10.6%) and 15 (7.6%) received HAV vaccine during the admission. Of the 174 (62.1%) patients at risk for HBV, ID recommended vaccination for 32 (18.3%) and 25 (14.4%) received HBV vaccine prior to discharge. 88 patients (31.4%) had no documentation of prior tetanus vaccination and ID recommended tetanus vaccination for 3 (1.1%). 5 patients (1.8%) received a tetanus booster during admission. In univariable analysis, the strongest factor associated with HAV (Odds Ratio 142.19, 95% Confidence Interval: 27.34-739.44) and HBV (Odds Ratio 18.98, 95% Confidence Interval: 7.02-51.37) vaccination during admission was ID recommendation for vaccine.

**Figure:**
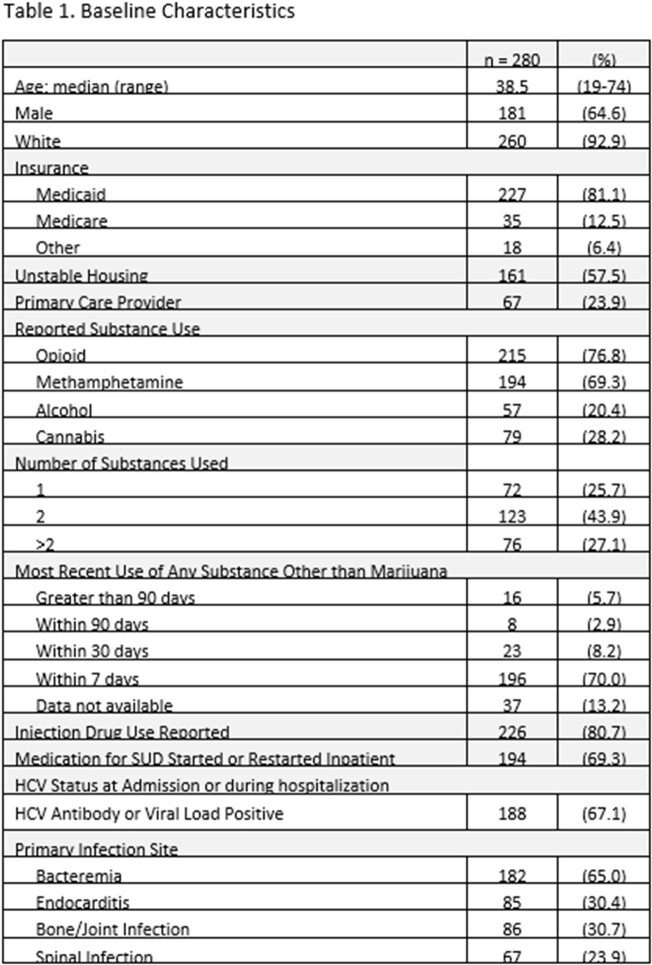

**Figure:**
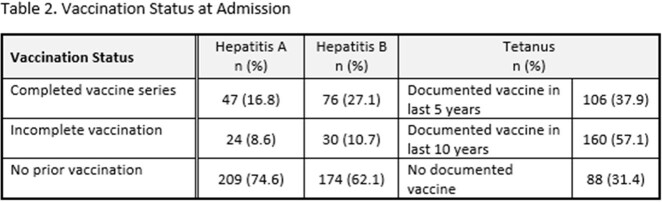

**Figure:**
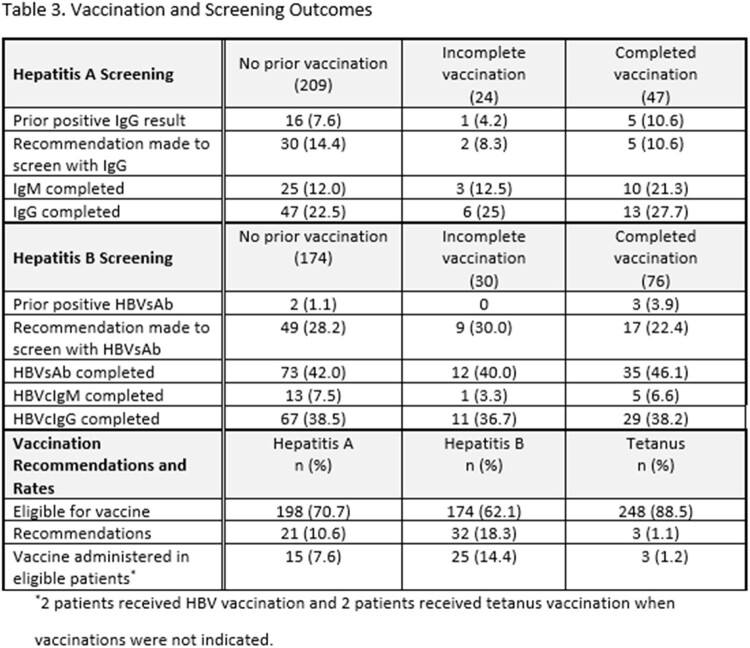

**Conclusion:**

Screening and vaccination for HAV and HBV remains low in the inpatient setting, as does tetanus vaccination in high-risk patients. A large proportion of our population (70%) were at risk for one or more of these preventable infections. Given the limited access and significant barriers to healthcare in this vulnerable population, efforts should be taken to maximize inpatient screening and vaccination for HAV, HBV, and tetanus.

**Disclosures:**

**Monica K. Sikka, MD**, F2G: Grant/Research Support

